# NBC update: The addition of viral and fungal databases to the Naïve Bayes classification tool

**DOI:** 10.1186/1756-0500-5-81

**Published:** 2012-01-31

**Authors:** Gail L Rosen, Tze Yee Lim

**Affiliations:** 1 Department of Electrical and Computer Engineering, Drexel University, Philadelphia, PA, USA; 2 Department of Physics, Drexel University, Philadelphia, PA, USA

## Abstract

**Background:**

Classifying the fungal and viral content of a sample is an important component of analyzing microbial communities in environmental media. Therefore, a method to classify any fragment from these organisms' DNA should be implemented.

**Results:**

We update the näive Bayes classification (NBC) tool to classify reads originating from viral and fungal organisms. NBC classifies a fungal dataset similarly to Basic Local Alignment Search Tool (BLAST) and the Ribosomal Database Project (RDP) classifier. We also show NBC's similarities and differences to RDP on a fungal large subunit (LSU) ribosomal DNA dataset. For viruses in the training database, strain classification accuracy is 98%, while for those reads originating from sequences not in the database, the order-level accuracy is 78%, where order indicates the taxonomic level in the tree of life.

**Conclusions:**

In addition to being competitive to other classifiers available, NBC has the potential to handle reads originating from any location in the genome. We recommend using the Bacteria/Archaea, Fungal, and Virus databases separately due to algorithmic biases towards long genomes. The tool is publicly available at: http://nbc.ece.drexel.edu.

## Background

While most metagenomics methods focus on identifying the prokaryotic content of a sample, fungi [1] and viral communities [2] play an important part in environmental communities. Therefore, it is of interest to classify fungal and viral sequences. There is no gene common to all viruses which makes metagenomics essential for understanding the viral component of environmental samples. Similar to the 16S rRNA gene of prokayrotes, the 18S rRNA gene is the short subunitRNA for fungi. However, it can be too highly conserved to distinguish fungal genera and species [3]. In addition, the large subunit (LSU) is also useful for fungi discrimination and is composed of the 5.8S and 26S-28S rRNA genes. Because the LSU may not contain enough information to distinguish fungal genera or species, the internal transcribed spacer (ITS) region has become useful due to its higher mutation rate. There are two ITS regions, ITS1 is between the 18S and the 5.8S genes, and ITS2 is between the 5.8S and the 26S-28S genes. The ITS region has commonly been targeted in recent studies [4,5].

Therefore, there is a need to classify viral and fungal sequences. All-in-one websites such as MG-RAST [6] contain viral databases in which to BLAST against. For fungi, traditionally, most sequences are classified using BLAST, with custom databases as the libraries of LSU and ITS sequences grow. For example, a pipeline has been developed to accelerate the process of ITS identification, where BLAST and alignment procedure has been customized for ITS sequences [7]. The University of Alaska at Fairbanks, offers a fast BLAST pipeline with LSU and ITS databases [8], and their ITS database is updated weekly at http://www.borealfungi.uaf.edu/. Recently, the well-known Ribosomal Database Project (RDP) has implemented an LSU classifier based on the näive Bayes approach [9] using a highly curated database developed by Los Alamos National Laboratories at http://rdp.cme.msu.edu/classifier.

With the advent of whole-genome sequencing studies, viruses and parts of the fungal genome that are not from the SSU, LSU, or ITS regions may need to be identified. Therefore, we introduce a viral and fungal database on NBC, to facilitate searches for genomic fragments. Unlike NBC's bacterial and viral databases which are categorized by strain [10], the fungal sequences are categorized by species for classification.

## Results and discussion

In the viral benchmarking, for the 45% of the viral segments originating from the database, the classifier had a 98% accuracy on the strain-level. For the 55% of segments that were novel viruses at the strain-level, the top-scoring organism was Acanthamoeba polyphaga mimivirus for 35% of the reads. This is due to the fact that the longer the genome is, the more combinations of sub-sequences it contains, called an N-mers. Since the NBC algorithm computes the likelihood of an N-mer given eachspecies [11], the longer the genome is, the more likely a novel query sequence will match to that genome by chance--giving longer genomes an advantage over shorter ones in the algorithm. This bias is due to the fact that in Bayes rule, *Pr*(*Genome*|*sequence*) = *Pr*(*sequence*|*Genome*)*Pr(*Genome*)/*Pr*(*Sequence*), the denominator Pr(Sequence) is considered equiprobable and can be ignored in the maximum likelihood calculation. This is not true but the true probability of the known sequence is unknown. This can be addressed with smoothing techniques, commonly found in the natural language processing literature [12]. The RDP classifier uses word-specific/genus-specific priors to calculate a "smoothing" estimate using frequencies found in the entire database. While this works well for more well-represented databases (such as RDP since it just contains 16S rRNA sequences), it will work less well for databases where less data is present. Therefore, we chose not to use a smoothing technique since most of our fungal sequences are only partial sequences and the viral database is highly incomplete. Also, such priors do not help the problem of query-sequence length normalization, which is a current issue with all classifiers. For repetitive sequences that may occur in fungal sequences, such sequences work to our advantage since the types of repeats vary among some species [13], and the NBC classifier can identify the species based on the type of repeat found in the query matching to the repetition in the database.

For novel viral segments where over 40% of the reads simulated from that virus matched to a genome that was not Acanthamoeba, we found 75% of them matched to the correct genera, and 78% of them matched to the correct order on average. Viruses that did not match to any one virus well or predominantly matched to Acanthamoeba were from orders and families, not represented in our viral database such as Tymovirales, Picornavirales, Reoviridae, and Retroviridae. A table of the viral segment originating organism, the number of reads generated from that organism, the predominant best match, and the percentage of reads match to that best match, is found in Additional File 1.

For the ITS dataset, we found that 54% of the reads which matched to NBC's top-hit species also match to the top-hit found by BLAST (from the study in [7]). Similary, 77% of the reads were from the same genera and 83% were from the same phylum. Most of the differences are due to lower-scoring reads against the BLAST database, which hit against an "uncultured fungus" or a general "fungus sp" in the NBC database. There were some cases such as Read 1371 which scored the best in BLAST to an uncultured endophytic fungus and where NBC's top hit was the second hit, Epicoccum. The results from Nilsson et al. with NBC's highest score match are found in Additional File 2.

For the LSU dataset, RDP and NBC are compared in Table 1. Since both methods are based upon the näive Bayes classifier, this is essentially a comparison of the databases underneath. Each classifier yields Ascomycota as the predominant Phylum, but NBC marks 10% less reads to this phylum. NBC yields more uncultured and unclassified fungi which results in 7.2% of the reads as having unknown phylum. RDP classifies the spurious 2% of reads that do not fit into Ascomycota and Basidiomycota into Fungi/Eukaryota incertae sedis, Chytridiomycota, Blastocladiomycota, and Glomeromycota. For the order level, NBC marks 36% of the reads as unknown. Yet, most of the most abundant orders have the same abundance in RDP and NBC. Differences in abundance are due to RDP yielding 7% more Capnodiales (a common indoor fungi) and 4% more Pleosporales (plant rot), and it has more orders such as Verrucariales and Polysporales (outdoor fungi). On the genus level, again RDP has many more taxa except for Glomerella, which causes anthracnose of wood and plants, in which NBC assigned 13% of the reads to. NBC also scores approximately 5% more reads as Aspergillus, which can be due to its simlarities to Eurotium. The results of each method on the LSU dataset, categorized by taxonomic level, can be found in Additional File 3 and Additional File 4.

**Table 1:** Distribution of the first 2000 reads of the Global House Dust fungal LSU dataset [14]

Phylum			Order			Genera		
	**RDP**	**NBC**		**RDP**	**NBC**		**RDP**	**NBC**

Ascomycota	82.2%	72.2%	Capnodiales	24.1%	17.6%	Cladosporium	11.1%	13.2%

Basidiomycota	16%	20.6%	Saccharomycetales/Endomycetales	13.4%	13.1%	Dermatocarpon	8.8%	

			Eurotiales/Elaphomycetales	9.6%	9.1%	Glomerella		13.0%

			Verrucariales	9%		Metschnikowia	8.7%	8.7%

			Pleosporales/Melanommatales	8%	3.9%	Eurotium	5.8%	

			Agaricales	5.5%	5.5%	Devriesia	3%	0.8%

			Hypocreales	3.6%	3.2%	Poria	3.3%	

			Polyporales	3.5%		Davidiella	3.1%	

			Dothideales	2.5%	1.8%	Alternaria	2.2%	1.6%

			Botryosphaeriales	1.6%	0.05%	Aspergillus	1.8%	6.6%

## Methods

We downloaded all completed viruses from http://ftp.ncbi.nih.gov/genomes/Viruses/ in January 2011, and we downloaded all fungal sequences in Genbank in April of 2010 and categorized them using their species label. We integrated these databases and taxonomic annotations on our website: http://nbc.ece.drexel.edu[10].

In order to test our classifier with the new databases, we used Metasim [15] to simulate Roche 454 sequencer reads from 2657 viral segments with approximately 100 reads generated per viral segment. The average read length of the simulated dataset was 272 bp, and the error model configuration contained 99 cycles (252 bp), lognormal distribution mean of 0.23 ± 0.15. The DNA clone parameters had normal distribution with mean of 2000 and second parameter of 200. Approximately, 55% of the test viral segments were not in the NBC database, because the Metasim viral dataset was a more updated version of the one that we had downloaded from NCBI for the NBC training data. We analyzed the two databases and determined that there was 55% novel strains in the MetaSim database compared to NBC's. We viewed this as an advantage in order to test how our classifier performs on viruses that were known and "novel", with respect to the database.

We did not simulate ITS and LSU segments using Metasim but acquired real datasets--a fungal ITS dataset from [7] and the first 2,000 reads from the LSU dataset of the global house dust project [14]. In Ley [16], it was shown that only 100 sequences per sample were sufficient to distinguish between mammalian guts. In further human studies [17], it is suggested that 1000 sequences/sample is a good trade-off between sampling depth and number of samples. Since we are sampling fungal sequences from dust, we overcompensate and take 2000 sequences. The main point is not to capture the actual diversity of the sample but to select a subset that can sufficiently compare the two tools (using the same sequences) and take less computational time--choosing 2000 sequences met this goal and is reasonable from the Ley and Hamady studies.

## Conclusions

NBC obtains similar results, especially at the family and order levels, to other competing classifiers. It should be noted that NBC has a bias towards longer genomes, so running a dataset against a mixture of different databases (e.g. Viral and Archaea/Bacteria) is discouraged. While NBC can take longer than some methods, it can be used for any type of sequence, provided that there is the training data for it. For example, Nilsson et al. [7] states their pipeline took 2 cpu-hours on the ITS dataset, ours took 4 cpu-hours. NBC is considerably slower than RDP, with the global house dust job taking 16 cpu-hours while running in minutes on RDP. This is due to the fact that NBC contains more data than just LSU genes, including ITS and whole-genomes. Unlike RDP, which only has a database for LSU, NBC has training data for LSU, ITS, and some fungal whole-genome sequences. To our knowledge, there are no published datasets where whole-genome fungal genomes have been sequenced. If the input reads are mixed fungal sequences (from ITS, LSU, and whole genome), NBC will give the same performance as if running two homogenous fungal datasets, since the classifier evaluates each read on an individual basis against the *whole *fungal database. Therefore, NBC speed is optimized for large sets of training data, and its drawback is that it runs slower than RDP. Unlike other classifiers, NBC's advantage is that it can be used for any type of sequence.

### Availability of supporting methods and data

The project home page is http://nbc.ece.drexel.edu. The ITS Fungal Sequences are available in Nilsson et al. [7]. The LSU and viral sequences are available on http://www.ece.drexel.edu/gailr/data.

## Competing interests

The authors declare that they have no competing interests.

## Authors' contributions

GR formulated the concept behind the project, tested the method, and wrote the paper. TYL built the fungal database. All authors read and approved the final manuscript.

## Supplementary Material

Additional file 1 **BC Results for Virus benchmarking**. Click here for file

Additional file 2 **Nilsson et al's BLAST results with our added NBC result for comparison, in Excel format**. Click here for file

Additional file 3 **NBC output for the Global House Dust sample, categorized by taxonomic level**. Click here for file

Additional file 4 **RDP output for the Global House Dust sample, categorized by taxonomic level**. Click here for file
